# A microprocessor-controlled film balance system

**DOI:** 10.1155/S1463924683000486

**Published:** 1983

**Authors:** R. Urhahn, P. Freyer, H. D. Ohlenbusch

**Affiliations:** Abteilung Physiologische Chemie der Medizinischen Fakultät der Rheinisch-Westfälischen Technischen Hochschule Aachen Neu-Klinikum Pauwelsstr. Aachen D 5100 Germany


Journal of Automatic Chemistry, Volume 5, Number 4 (October–December 1983), pages 197–200

### A microprocessor-controlled film balance system

**R. Urhahn, P. Freyer and H. D. Ohlenbusch**

*Abteilung Physiologische Chemie der Medizinischen Fakultät der Rheinisch-Westfälischen Technischen Hochschule Aachen, Neu-Klinikum Pauwelsstr., D 5100 Aachen, FR Germany*

### Introduction

Film balances are widely used to study the physical and chemical properties of monomolecular films of surface-active substances, such as lipids and proteins at the gas-liquid interface. Usually film balances measure the two-dimensional surface pressure, Π, as a function of the molecular area, *A*, at constant temperature (Π-*A* isotherm). Some further developments and modifications of film balances have recently been announced [1–4]. Most of these record the film data by means of an X-Y recorder after manual spreading of the film substance. This procedure has several disadvantages. Further manipulations of the analogically recorded data, for example plotting them in other diagrams, calculating derivatives or fitting theoretical curves, are very tedious and time-consuming operations. Also, essential experimental conditions such as the surface-pressure stability cannot automatically be checked in order to avoid artifacts caused by drift of the measuring system. It is desirable to have an automatic spreading procedure integrated into the control of the whole experiment.

In order to optimize the control of film balance measurements and to facilitate the analysis of the results a micro-processor control unit was developed for a conventional film balance. In addition, a controllable spreading device for the delivery of spreading solutions to the surface was constructed. The design and performance of the control unit combined with the spreading device and a film balance are the subject of this article.

### System design

Figure 1 shows the configuration of the system. The control unit is placed in a protective, easy-to-use cabinet (dimensions 30 x 36 x 3 cm). The unit is based on a microprocessor-development kit—Intel SDK-85. EPROM and RAM have been expanded, respectively, to 4k and to 8k. The RAM is backed up against power failure by rechargeable batteries. In addition, the control unit is provided with a programming device for EPROMs and a quartz-triggered timer. Parallel and serial I/O interfaces connect the control unit to the film balance, the spreading device, a printer and a host computer (the CDC Cyber 175).

The film balance is an unchanged, commercially available, analogically operating model of the Langmuir type (MGW Lauda). The trough with measuring and moving barrier is schematically shown in figure 1. All the functions of this model, such as the control of the moving barrier (start/stop, expansion/compression), are activated by the control unit via relays. Manual operation, however, is still possible if no microprocessor control is desired. Two digital voltmeters with BCD output (Analogic 2575) make the Π and *A* proportional voltage outputs of the Lauda model available to the control unit in digital form.

The spreading device consists of a stepper motor (Berger, RDM 50/12), a control chip (Landis & Gyr, SAA 1042) and a gas-tight microsyringe (Hamilton). Motor and syringe are linked by a gear. The gear ratio was chosen so that low flow rates, which are essential for the spreading of protein solutions, can be obtained. The control chip receives the direction of rotation and the trigger pulses from the control unit and converts them to appropriate driving pulses for the stepper motor. Hamilton microsyringes of different volumes can be used. Spreading solutions are usually deposited directly onto the liquid surface from a height of a few millimeters.

The printer (Kontron, OEM 5010) records the measured values and the control parameters of the spreading and measuring procedure. For individual manipulations, such as curve fitting, plots and calculation of surface thermodynamic quantities the stored data are transferred via serial I/O (RS 232C) to a host computer. For the control of additional devices the I/O capacity can easily be expanded.

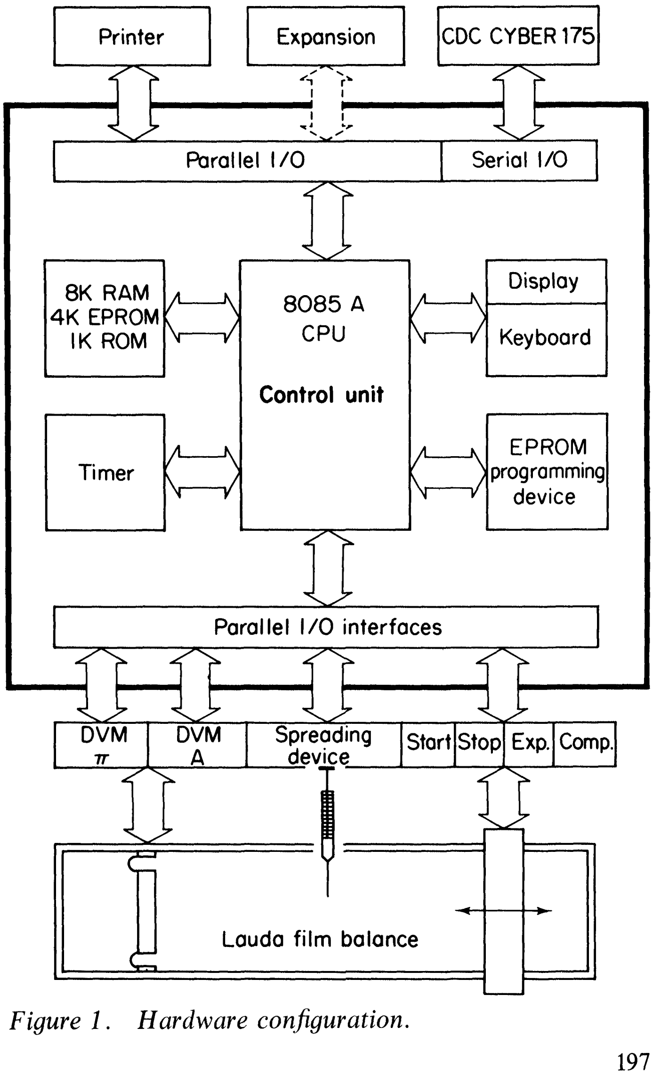

*Figure 1. Hardware configuration.*

### Control software and system performance

All programs are written in the Intel 8080/85 compatible Z80 assembler language. They are assembled by a host computer-resident Fortran cross-assembler (Mostek, modified XFOR80), transmitted via serial I/O to the control unit and then programmed into EPROMs by means of the EPROM programming device.

The system's software can be divided into two sections. The first contains the SDK-85 monitor (Intel) for communication via keyboard/display, programs for EPROM programming and data transfer, and several utility routines. The second section, which consists of the spreading and measuring programs including the subroutines for the direct control of the DVMs, the film balance functions, the spreading device and the printer, is briefly outlined below.

The spreading program controls the delivery of spreading solutions to the surface. The flow rate is determined by the control parameter 'volume' and 'spreading time'. Using a 1 ml microsyringe, for example, the flow rate can be varied from 0·01 ml/min to 0·2 ml/min. Several measuring programs have been developed; two expandable basic versions (P1 and P2) are illustrated in figures 2 and 3. The data-acquisition procedure is similar in both programs. For example $\Pi$ is stored and printed only if the absolute value of the difference between the actual and last stored value $|\Pi - \Pi'|$ exceeds the control parameter $\Delta\Pi$.

Program P1 (figure 2) records the surface pressure, $\Pi$, as a function of the molecular area, A, by means of compression/expansion with the moving barrier. Usually, the experiment starts with the compression. In addition, the program records the time, $t$, for later calculation of compression/expansion rates. The program starts with the recording of the first values, in the course of which $\Pi$ is repeatedly read and then averaged. Subsequently, the values ($\Pi$, $A$, $t$) are printed and stored in the RAM. The next acquisition is controlled by the parameters $\Delta\Pi$ and $\Delta$A. After reaching the programmed limits (maximum $\Pi$ and minimum $A$) the direction of the moving barrier is changed and further runs are possible.

Program P2 (figure 3) maintains $\Pi$ at a constant level and records the area $A$ as a function of time $t$. It is used to study time-dependent processes at the interface—adsorption and desorption of molecules for example. The surface pressure is held at $\Pi_c$ $\pm p$ by means of the moving barrier; $\Pi_c$ is the programmed surface pressure and $p$ is a control parameter. Data acquisition is controlled by the parameters $\Delta A$ and $\Delta t$. The program is terminated if the actual area reaches a minimum value.

The operator communicates with the system via display and keyboard, which serves for all manipulations, such as start/stop of programs and the input of the control parameters. Figure 4 illustrates the operating procedure. Initially the system looks for free RAM space and writes a BOF mark (beginning of file). The operator enters the parameter for the control of the film balance and spreading device and the data file label—experiment number, concentration of spreading solution and calibration factors. After checking the stability of $\Pi$ the moving barrier is set to initial position and the spreading program is called. After

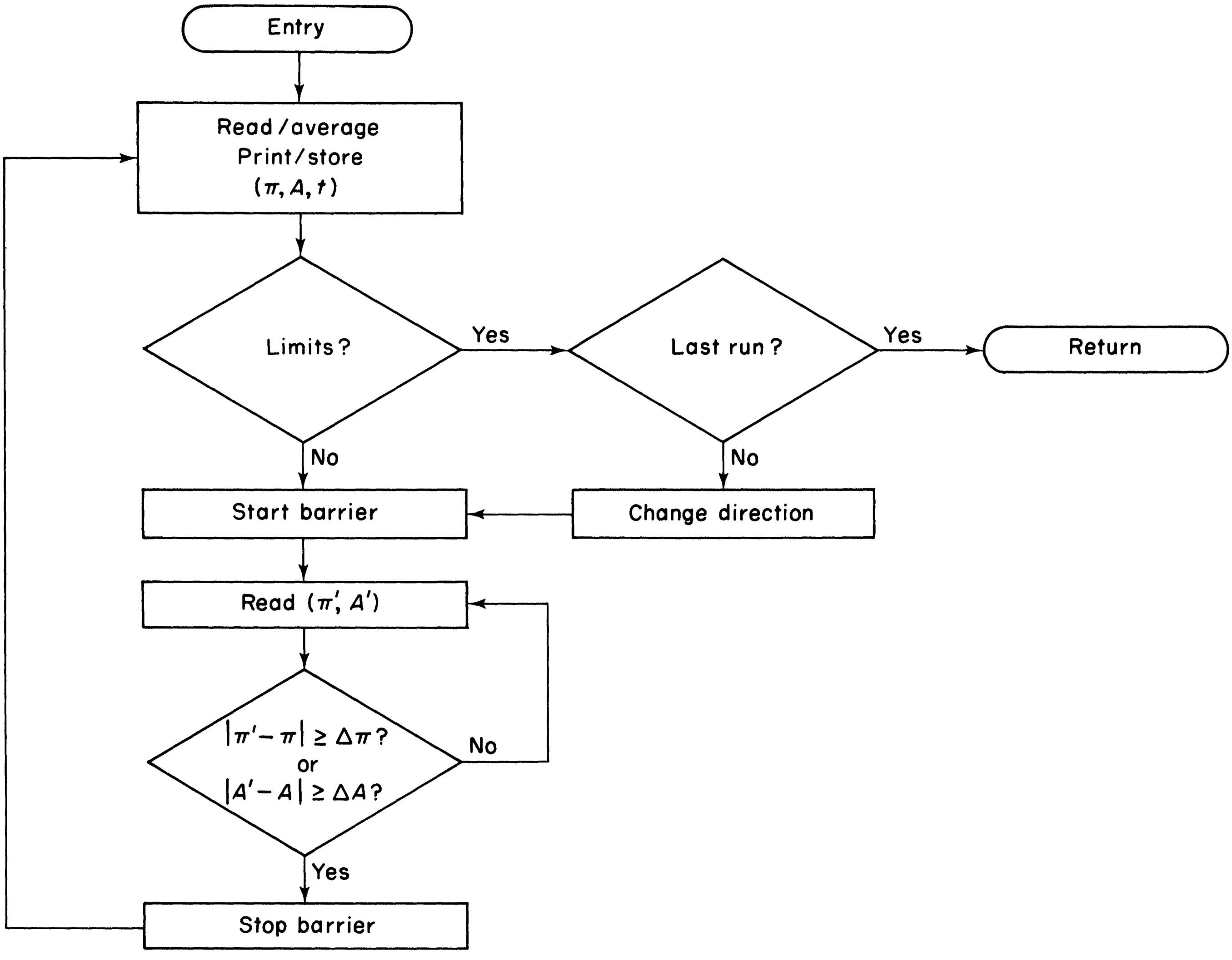

*Figure 2. Flow chart for recording* $\Pi$-$A$ *isotherms (P1).*

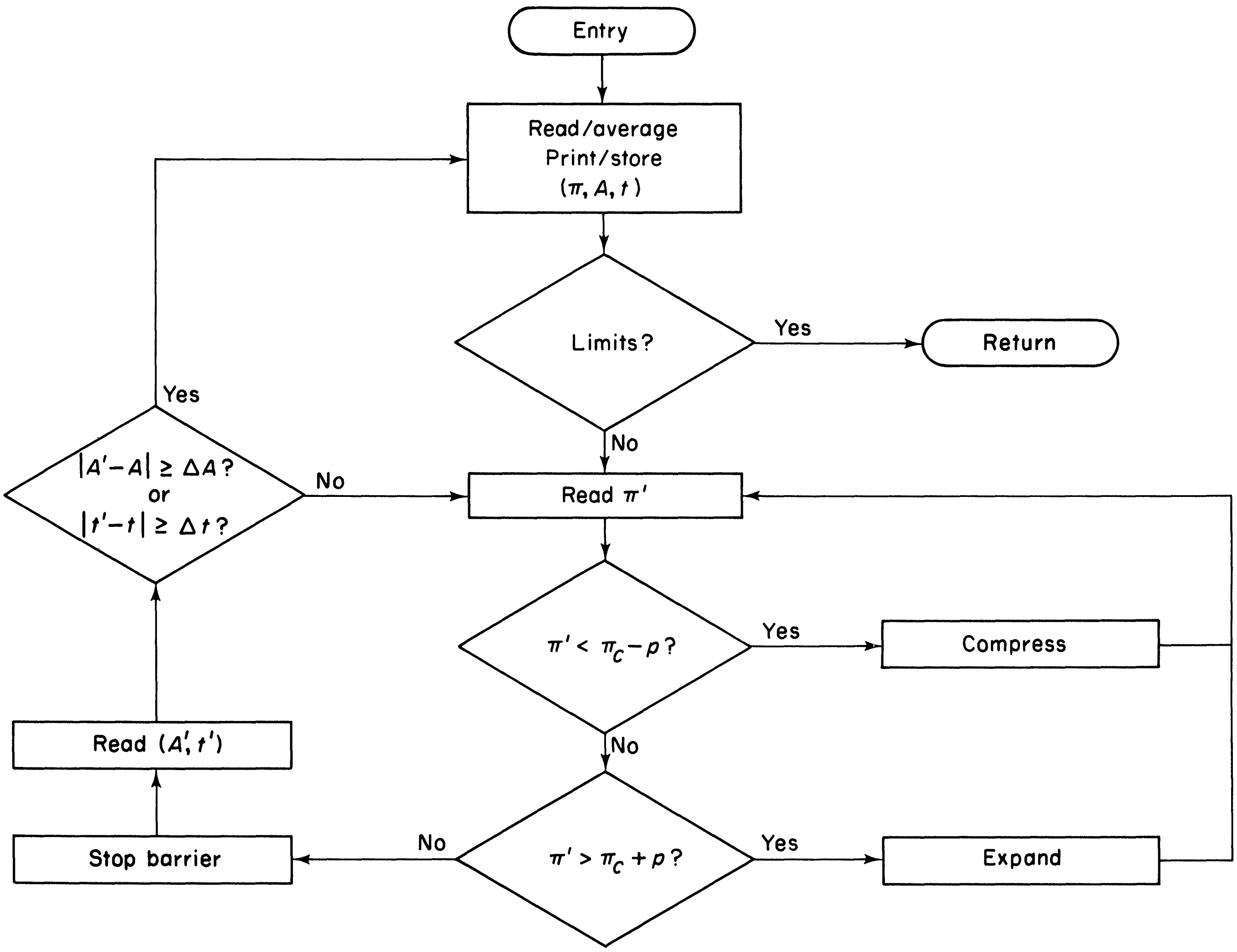

*Figure 3. Flow chart for recording $A(t)$ at $\Pi$ = constant (P2).*

complete spreading the system starts the timer and calls the desired measuring program. Data acquisition is closed by an EOF mark (end of file). Having completed a series of experiments the data files are transferred to a host computer for individual manipulations.

### Results

In order to test the system measurements were carried out with oleic acid (Serva, Stock No. 31069 p.A.) dissolved in petroleum ether (Merck, Stock No. 1774 p.A.). Figure 5 shows a plot of the surface pressure versus area/molecule (oleic acid on 0·01 M HCl at 21°C [concentration: 0·258 mg/ml, spreading volume: 0·180 ml, flow rate: 0·100 ml/min, compression rate: 0·75 $\times 10^{-2}$nm$^2$/molecule $\times$ min]; the inset is a plot of surface compressibility versus molecular area). The surface pressure drift was less than 0·1 mN/m $\times$ h). For the accurate determination of the area at the point of collapse, the surface compressibility, $-1/A(\Delta A/\Delta\Pi)$, was calculated from the measured $\Pi$-$A$ values. A plot of the compressibility versus area/molecule (inset) yields a value of $28 \times 10^{-2}$nm$^2$/molecule which agrees with the literature [5].

The system is presently being used for the investigation of protein monolayers.

### Conclusion

The microprocessor control unit is a powerful low-cost supplement to a conventional film balance. It offers flexible control of film balance experiments and allows individual data analysis. The spreading device enables a precise delivery of spreading solutions to the surface. By proper combination and modification of the programs the user is able to adapt the control of film balance and spreading device to experimental conditions in an optimal way.

With few modifications the control unit can be connected to other film balances. (Programs and electronic circuit diagrams are available from the authors on request.) The film balance and spreading device combination can be used for parallel control of other instruments which investigate surfaces—such as surface potential meters or preparative devices for handling multilayers.

### Acknowledgements

The authors wish to thank H. Szameit and J. Phlippen for their technical assistance and G. Bennin for making the figures.

### References

1. Abraham, B. M., Miyano, K., Buzard, K. and Ketterson, J. B., *Review of Scientific Instruments*, **51** (1980), 1083.
2. Albrecht, O. and Sackmann, E., *Journal of Physics E: Scientific Instruments*, **13** (1980), 512.
3. Munger, G. and Leblanc, R. M., *Review of Scientific Instruments*, **51** (1980), 710.
4. Stenberg, M. and Löfgren, H., *Review of Scientific Instruments*, **50** (1979), 1098.
5. Markley, K. S., *Fatty Acids—Part 1* (Interscience, New York, 1960), 629.

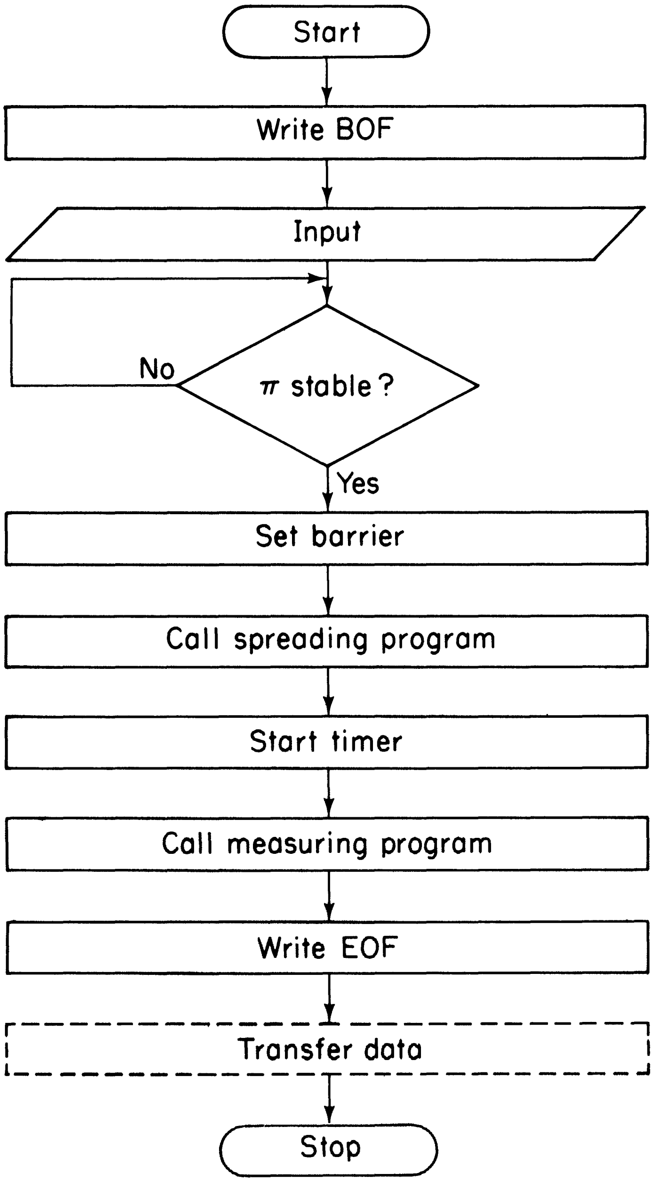

*Figure 4. Operating procedure.*

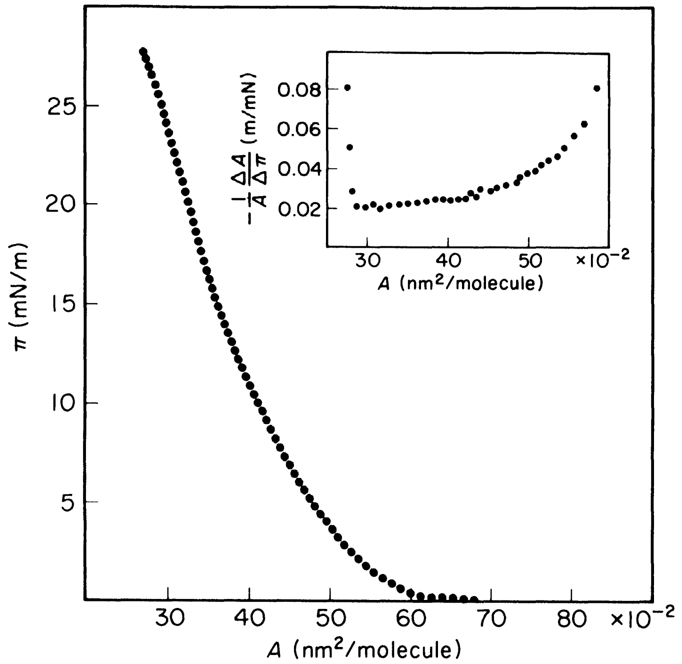

*Figure 5. Plot of surface pressure versus molecular area.*

### NOTES FOR AUTHORS

*Journal of Automatic Chemistry* covers all aspects of automation and mechanization in analytical, clinical and industrial environments. The *Journal* publishes original research papers; short communications on innovations, techniques and instrumentation, or current research in progress; reports on recent commercial developments; and meeting reports, book reviews and information on forthcoming events. All research papers are refereed.

### Manuscripts

Two copies of articles should be submitted to the Editor. All articles should be typed in double spacing with ample margins, on one side of the paper only. The following items should be sent: (1) a title-page including a brief and informative title, avoiding the word 'new' and its synonyms; a full list of authors with their affiliations and full addresses; (2) an abstract of about 250 words—this should succinctly describe the scope of the contribution and highlight significant findings or innovations; it should be written in a style which can easily be translated into French and German; (3) the main text with sections and subsections numbered; (4) appendices (if any); (5) references; (6) tables, each table on a separate sheet and accompanied by a caption; (7) illustrations (diagrams, drawings and photographs) numbered in a single sequence from 1 upwards and with the author's name on the back of every illustration; captions to illustrations should be typed on a separate sheet. Papers are accepted for publication on condition that they have been submitted only to *Journal of Automatic Chemistry*.

### References

References should be indicated in the text by numbers following the author's name, i.e. Skeggs [6]. In the reference section they should be arranged thus:

to a journal

MANKA, D. P., *Journal of Automatic Chemistry*, **3** (1981), 119.

to a book

MALMSTADT, H. V., in *Topics in Automatic Chemistry*, Ed. Stockwell, P. B. and Foreman, J. K. (Horwood, Chichester, 1978), p. 68.

### Illustrations

Line diagrams are preferred to photographs. Original copies of diagrams and drawings should be supplied, and should be drawn to be suitable for reduction to the page or column width of the *Journal*, i.e. to 85 mm or 179 mm, with special attention to lettering size. Photographs may be sent as glossy prints or as negatives.

### Proofs and offprints

The principal or corresponding author will be sent galley proofs for checking and will receive 50 offprints free of charge. Additional offprints may be ordered on a form which accompanies the proofs.

*Manuscripts should be sent to the Editor: Dr Peter B. Stockwell, P.S. Analytical Ltd, 2 Eagles Drive, Tatsfield, Westerham, Kent TN16 2PB, UK.*

